# Intracellular ATP does not inhibit Slo2.1 K^+^ channels

**DOI:** 10.14814/phy2.12118

**Published:** 2014-09-11

**Authors:** Priyanka Garg, Michael C. Sanguinetti

**Affiliations:** 1 Nora Eccles Harrison Cardiovascular Research & Training Institute, University of Utah, Salt Lake City, Utah, USA; 2 Department of Pharmaceutics and Pharmaceutical Chemistry, University of Utah, Salt Lake City, Utah, USA; 3 Department of Internal Medicine, Division of Cardiovascular Medicine, University of Utah, Salt Lake City, Utah, USA

**Keywords:** ATP, KCNT2, oocytes, potassium channels, *Xenopus*

## Abstract

Under normal physiological conditions, the open probability of Slo2.1 K^+^ channels is low. Elevation of cytosolic [Na^+^] and [Cl^−^] caused by ischemia or rapid electrical pacing of cells increases the open probability of Slo2.1 channels and the resulting outward current can stabilize the resting state of cells. Initial characterization of heterologously expressed human Slo2.1 indicated that these channels were inhibited by physiological levels of intracellular ATP. However, a subsequent study found that intracellular ATP had no effect on Slo2.1 channels. Here, we re‐examine the effects of intracellular ATP on cloned human Slo2.1 channels heterologously expressed in *Xenopus* oocytes. Our studies provide both direct and indirect evidence that changes in intracellular [ATP] have no effect on Slo2.1 channels. First, we directly examined the effects of intracellular ATP on Slo2.1 channel activity in excised inside‐out macropatches from *Xenopus* oocytes. Application of 5 mmol/L ATP to the intracellular solution did not inhibit Slo2.1 currents activated by niflumic acid. Second, we lowered the [ATP]_i_ in whole oocytes using the metabolic inhibitor NaN_3_. Depletion of [ATP]_i_ in oocytes by 3 mmol/L NaN_3_ rapidly activated heterologously expressed K_ATP_ channels, but did not increase wild‐type Slo2.1 channel currents activated by niflumic acid or currents conducted by constitutively active mutant (E275D) Slo2.1 channels. Third, mutation of a conserved residue in the ATP binding consensus site in the C‐terminal domain of the channel did not enhance the magnitude of Slo2.1 current as expected if binding to this site inhibited channel function. We conclude that Slo2.1 channels are not inhibited by intracellular ATP.

## Abstract (precis)

Initial characterization of heterologously expressed human Slo2.1 indicated that these channels were inhibited by physiological levels of intracellular ATP. Here, we re‐examine the effects of intracellular ATP on cloned human Slo2.1 channels heterologously expressed in Xenopus oocytes. Our studies provide both direct and indirect evidence that changes in intracellular [ATP] have no effect on Slo2.1 channels.

## Introduction

Intracellular Na^+^‐activated K^+^ (K_Na_) channels were first described in guinea pig cardiomyocytes 30 years ago (Kameyama et al. 1984) and later in cultured trigeminal ganglion neurons from quail embryos (Bader et al. 1985; Haimann et al. 1990). K_Na_ channels in neurons can either be activated by Na^+^ ions that are conducted by voltage‐activated Na^+^ channels during the rapid upstroke phase of action potentials (Bader et al. 1985) or by a persistent inward Na^+^ current (Budelli et al. 2009). However, in the heart it appears that K_Na_ channels are only activated under pathological conditions such as hypoxia or ischemia (Kameyama et al. 1984). The molecular basis of K_Na_ channels was discovered in 2003 with the cloning of Slo2.1 (Bhattacharjee et al. 2003) and Slo2.2 (Yuan et al. 2003). Based on RT‐PCR, Slo2.1 is expressed in most tissues, including the brain, heart, skeletal muscle, lung and liver, whereas Slo2.2 is highly expressed within specific regions of the brain, and to a lesser extent in the kidney, testis, and very weakly in the heart (Yuan et al. 2003). The Slo2.1 channel was first described as a functional hybrid between a K_Na_ and K_ATP_ channel because it was activated by intracellular Na^+^ (and Cl^−^) and inhibited by intracellular ATP (Bhattacharjee et al. 2003). Activation of large conductance Slo2.1 K^+^ channels in response to elevated [Na^+^]_i_ and depletion of intracellular ATP as occurs during hypoxia or ischemia might protect cardiomyocytes or neurons from damage such as Ca^2+^‐overload by stabilizing the resting (hyperpolarized) state of the cell.

Consistent with its proposed sensitivity to changes in [ATP]_i_, Slo2.1 channels contain a consensus ATP binding site just C‐terminal to the second cytoplasmic RCK (regulate conductance of K^+^) domain. Inhibition of Slo2.1 currents by intracellular ATP (~80% reduction at 5 mmol/L) in whole cells or excised membrane patches of CHO cells transfected with *Slo2.1* cDNA was reported to be dependent on the integrity of this motif (Bhattacharjee et al. 2003). However, subsequent studies on striatal interneurons and HEK293T cells transfected with *Slo2.1* cDNA showed no effect of intracellular ATP on Slo2.1 channel activity (Berg et al. 2007), consistent with the early studies of cardiac K_Na_ channels (presumably Slo2.1) that were recorded in the presence of 2 mmol/L K_2_ATP in the intracellular solution (Kameyama et al. 1984). Moreover, we have found that heterologously expressed human Slo2.1 channels can be robustly activated by either niflumic acid (NFA) or elevation of [NaCl]_i_ (Dai et al. 2010; Garg et al. 2013; Garg and Sanguinetti 2012) in intact *Xenopus* oocytes where [ATP]_i_ is estimated to be 4.6 mmol/L (Gribble et al. 2000). As inhibition of Slo2.1 by intracellular ATP has been cited as one of the defining features of these channels (Bhattacharjee and Kaczmarek 2005; de Los Angeles Tejada et al. 2012; Garg et al. 2013; Garg and Sanguinetti 2012; Kaczmarek 2013; Paulais et al. 2006), in this study we have investigated the effects of altering cytosolic [ATP] on heterologously expressed human Slo2.1 channels in intact *Xenopus* oocytes and in excised inside‐out macropatches.

## Materials and Methods

### Molecular biology

cDNA for human *Slo2.1* (*KCNT2*, NCBI Genbank accession no. NM_198503), kindly provided by L. Kaczmarek (Yale University), was subcloned into the psGEM oocyte expression vector as described (Dai et al. 2010). K1031A Slo2.1 was generated by using the QuikChange site‐directed mutagenesis kit (Agilent Technologies) and confirmed by DNA sequencing by the University of Utah sequencing core facility. Human *Kir6.2* (*KCNJ11*) and rat *SUR1* (*ABCC8*) cDNAs in the pBF vector were kindly provided by F. Ashcroft (University of Oxford). Plasmids were linearized with SfiI (*Slo2.1*) or Mlu1 (*Kir6.2*,* SUR1*). cRNAs were prepared using the mMessage mMachine T7 kit (*Slo2.1*) or SP6 kit (*Kir6.2*,* SUR1*) (Ambion, Life Technologies, Grand Island, NY). Concentrations of cRNA were measured with the RiboGreen RNA quantitation kit (Invitrogen, Life Technologies).

### Oocyte isolation and cRNA injection

Methods for isolation of oocytes from *Xenopus laevis* were approved by the Institutional Animal Care and Use Committee of the University of Utah. Briefly, adult female frogs were anesthetized by immersion in a 0.2% tricaine methanesulfonate solution. A small surgical incision was made to remove the ovarian lobes. To separate the individual oocytes from follicle cells, ovarian lobes were manually dispersed using tweezers and placed into a Ca^2+^‐free saline solution containing 2 mg/ml each of type I and II collagenase (Worthington Biochemical Corporation, Lakewood, NJ) and gently shaken for 1–1.5 h. The Ca^2+^‐free saline solution contained (in mmol/L): 96 NaCl, 2 KCl, 1 MgCl_2_, and 5 HEPES; pH was adjusted to 7.6 with NaOH.

To study *I*_Slo2.1_, stage IV and V oocytes were injected with 1 ng *Slo2.1* cRNA for whole cell recordings or 15 ng cRNA for excised‐patch recordings. To study ATP‐sensitive K^+^ channel current (*I*_KATP_), oocytes were injected with 25 ng *Kir6.2* and 46 ng *SUR1* cRNA. Injected oocytes were incubated at 18°C for 1–7 days (Slo2.1) or 3 days (Kir6.2/SUR1) in Barth's saline solution before use in voltage clamp experiments. Barth’ solution contained (in mmol/L): 88 NaCl, 1 KCl, 0.41 CaCl_2_, 0.33 Ca(NO_3_)_2_, 1 MgSO_4_, 2.4 NaHCO_3_, 10 HEPES, and 1 pyruvate plus gentamycin (50 mg/L); pH was adjusted to 7.4 with NaOH.

### Two‐electrode voltage‐clamp protocol

Standard two‐electrode voltage‐clamp (TEVC) techniques (Goldin 1991; Stuhmer 1992) were used to record whole cell Slo2.1 K^+^ channel current (*I*_Slo2.1_) at room temperature. Oocytes were placed in a small chamber (0.3 mL volume, RC‐1Z; Warner Instruments) and superfused at a rate of ~2 mL/min with recording solution that contained (in mmol/L): 98 NaCl, 2 KCl, 1 CaCl_2_, 1 MgCl_2_, and 5 mmol/L HEPES; pH adjusted to 7.6 with NaOH. To study *I*_KATP_, this solution was modified by omitting NaCl and increasing the [KCl] to 104 mmol/L.

Agarose‐cushion microelectrodes used for TEVC recordings were fabricated as described previously (Schreibmayer et al. 1994) and had tip resistances ranging from 0.2 to 0.8 MΩ after back‐filling with 3 M KCl. A Dell 2400 computer, GeneClamp 500 amplifier, Digidata 1322A data acquisition system, and pCLAMP 8.2 or 9.0 software (Molecular Devices, Sunnyvale, CA) were used to produce voltage protocols and to record digitized current and voltage signals.

### Macropatch recordings

Multi‐channel patch recordings of *I*_Slo2.1_ were performed using the standard excised inside‐out configuration of the patch clamp technique (Hamill et al. 1981). The vitelline membrane from each oocyte was removed manually after treatment with hypertonic solution (400 mmol/L sucrose) for 2–6 min. Patch pipettes were fabricated from 1 mm OD borosilicate glass capillaries (World Precision Instruments, Sarasoto, FL) using a Sutter Instruments P‐97 puller (Sutter Instrument, Novato, CA). Pipette tips were heat polished after pulling and had a resistance of 2–4 MΩ when filled with a pipette (extracellular) solution that contained (in mmol/L): 90 NaCl, 10 KCl, 1 CaCl_2_, 1 MgCl_2_, 10 HEPES and 2 niflumic acid (NFA); pH was adjusted to 7.2 with NaOH. NFA was added to the pipette solution to activate *I*_Slo2.1_. The bath (intracellular) solution contained (in mmol/L): 90 KCl, 10 NaCl, 2 EGTA, 2 MgCl_2_, 10 HEPES and 10 μM phosphatidylinositol 4,5‐bisphosphate diC8 (PI(4,5)P_2_ diC8); pH was adjusted to 7.2 with KOH. During the day of recording, the stock solution of PI(4,5)P_2_ diC8 was kept on ice until just before use when it was added to the intracellular solution.

A Dell computer, Axopatch 200B patch clamp amplifier, Digidata 1322A data acquisition system and pCLAMP 9.0 software (Molecular Devices) were used to produce command voltage steps and to record currents. Currents were digitized at 5 kHz after on‐line filtering at 1 kHz with an eight‐pole low‐pass Bessel filter. After patch excision, macroscopic *I*_Slo2.1_ was recorded for at least 4 min to ensure stability before 5 mmol/L K_2_ATP was added to the intracellular solution. Currents were recorded during 1‐s voltage ramps applied from +80 to −140 mV. The holding potential was −80 mV and the interval between voltage ramps was 5 s.

### Data analysis

pCLAMP9 (Molecular Devices) and Excel (Microsoft, Redmond, WA) software were used to analyze digitized data, and Origin 8.5 (OriginLab, Northampton, MA) was used to prepare figures. Results are expressed as mean ± SEM. (*n* = number of oocytes). Statistical analysis was performed using Prism 5 software (GraphPad Software, Inc., La Jolla, CA). Statistical significance was evaluated using paired Student's *T*‐test or ANOVA (one‐way or two‐way) with Tukey's multiple comparison post‐hoc test where appropriate. A *P*‐value <0.05 was considered significant.

### Chemicals

Sodium azide (NaN_3_), niflumic acid (NFA) and K_2_ATP were purchased from Sigma‐Aldrich (St. Louis, MO). PI(4,5)P_2_ diC8 (Echelon Biosciences Inc., Salt Lake City, UT) was dissolved in the macropatch intracellular solution and stored in glass vials as a 230 μM stock at −80°C. All working solutions were prepared immediately before use by dilution of stock solutions previously stored at −20°C.

## Results

### Intracellular ATP has no effect on *I*_Slo2.1_ recorded from inside‐out macropatches of oocyte membrane

**Figure 1.**
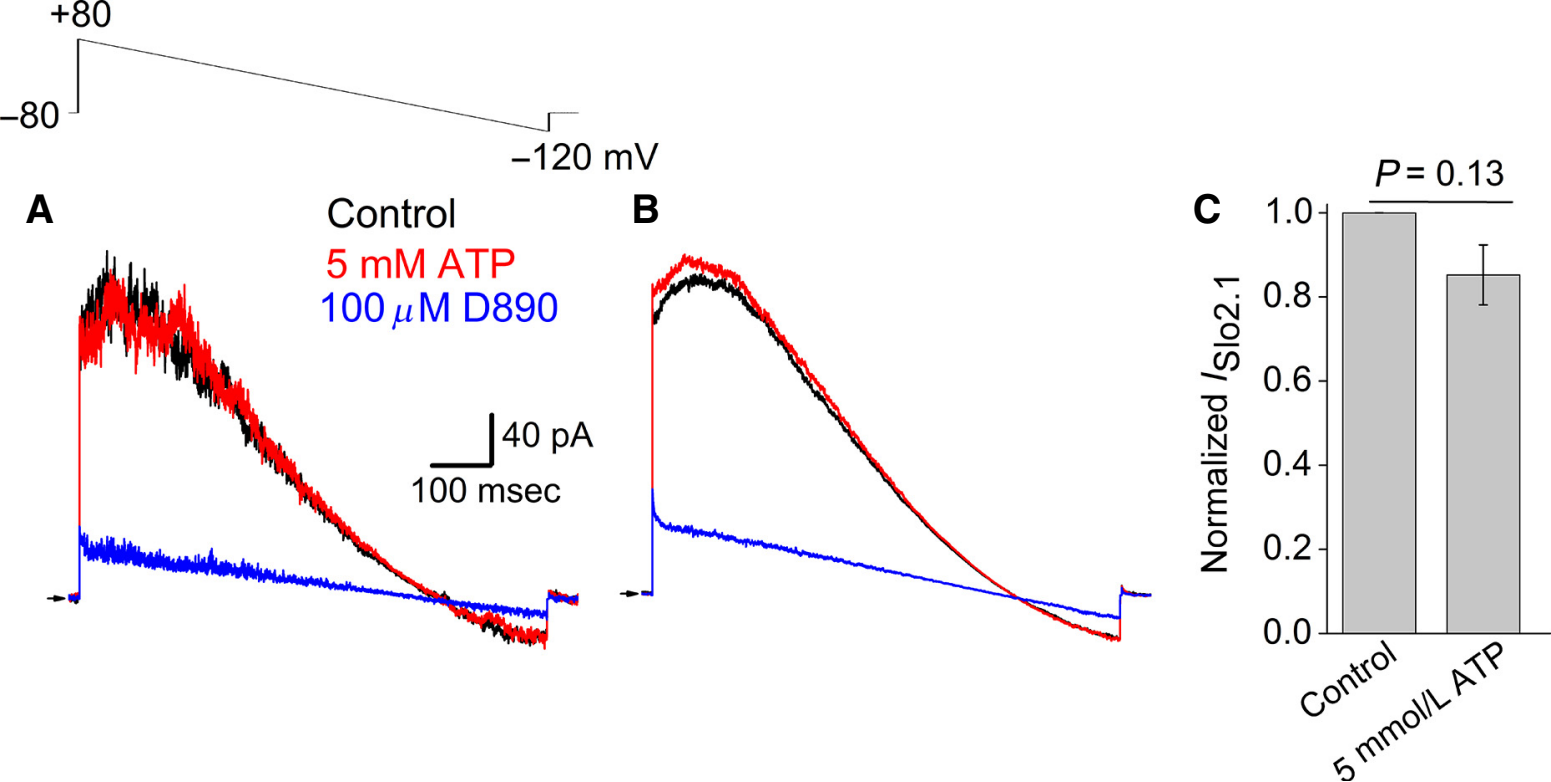
Intracellular ATP does not inhibit *I*_Slo2.1_ recorded from inside‐out macropatches of *Xenopus* oocyte membranes. (A) Representative current traces recorded from excised inside‐out macropatches of oocyte membrane during voltage ramps (upper inset) from +80 to −120 mV. Currents were recorded in the absence (Control, black trace), 5 min after addition of 5 mmol/L K_2_ATP (red trace), and after addition of 100 μM D890 in the continued presence of K_2_ATP. Arrow indicates 0 current level. (B) Mean currents from 30 to 35 consecutive sweeps under each condition as indicated. (C) Bar graph comparing normalized peak *I*_Slo2.1_ in the absence and presence of 5 mmol/L K_2_ATP. No statistical difference was observed between the two groups (paired Student's t test; *P *= 0.13, *n *= 4).

The effect of ATP applied directly to the intracellular side of inside‐out membrane patches excised from *Xenopus* oocytes was determined. In most patches, the magnitude of *I*_Slo2.1_ decreased rapidly soon after patch excision, complicating analysis of the effects of intracellular ATP. However, the rate of current run‐down was greatly reduced by including 10 μM PI(4,5)P_2_ diC8 to the solution bathing the inside (cytosolic side) of the membrane patch. Slo2.1 channels can be activated by elevated [NaCl]_i_ delivered via intracellular pipettes in intact oocytes (Garg et al. 2013). However, Slo2.1 channels were not activated in excised inside‐out macropatches when [NaCl]_i_ was elevated from 3 mmol/L to 30 or 300 mmol/L, even in the presence of 10 μM PI(4,5)P_2_ diC8. Therefore, heterologously expressed Slo2.1 channels were activated by 2 mmol/L NFA in the pipette solution. *I*_Slo2.1_ was recorded under voltage clamp in response to repetitive ramping of the transmembrane voltage from +80 to −120 mV (Fig. 1A, inset). Under control conditions, large outward currents were activated almost instantly in response to stepping the voltage from −80 mV to +80 mV, and current magnitude declined as the voltage was ramped to −120 mV, becoming inward at potentials negative to −80 mV (Fig. 1A, black trace). After several voltage ramps were applied, 5 mmol/L K_2_ATP was added to the intracellular solution and the voltage ramps were continued for another 5 min. There was no difference between the currents recorded in the presence of 5 mmol/L ATP (Fig. 1A, red trace) compared to the control trace. Currents were rapidly and almost fully blocked by addition of 100 μM *N*‐methyl verapamil (D890) to the intracellular solution (Fig. 1A, blue trace), confirming that the membrane patch was in the inside‐out configuration as the permanently charged D890 only blocks *I*_Slo2.1_ when added to the intracellular side of the membrane (Garg et al. 2013). Figure 1B shows currents from the same patch after averaging multiple traces under each condition. The effect of 5 mmol/L K_2_ATP was determined in a total of 4 patches, and no significant change in *I*_Slo2.1_ was detected (Fig. 1C).

### NaN_3_ activates ATP‐sensitive K^+^ (K_ATP_) channels, but not Slo2.1 channels

**Figure 2.**
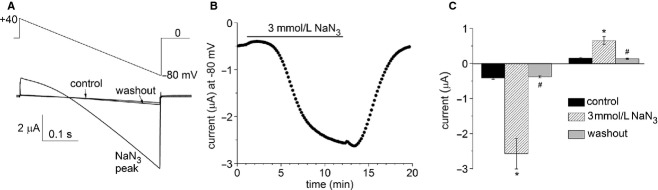
NaN_3_ activates *K*_ATP_ channels heterologously expressed in *Xenopus* oocytes. (A) Voltage ramp protocol (upper panel) used to elicit whole‐cell currents (lower panel) measured by TEVC. Currents were recorded under control conditions, after 10 min exposure to 3 mmol/L NaN_3_ and after washout of NaN_3_. Arrow indicates 0 current level. (B) Time‐dependent changes in the inward current recorded at −80 mV in a single representative oocyte induced by application and subsequent washout of 3 mmol/L NaN_3_. (C) Bar graphs comparing *I*_KATP_ measured at −80 and +40 mV during voltage ramps before (control), at peak of NaN_3_ effect and after washout of NaN_3_ (*n *= 10). *P < 0.001 relative to control, ^#^*P *< 0.001 relative to NaN_3_‐peak current (one‐way ANOVA and Tukey's multiple comparison test).

The properties of *I*_Slo2.1_ may be altered in excised membrane patches due to stretching of the membrane and exchange of the complex contents of cytoplasm with a simple intracellular salt solution. To determine if changes in [ATP]_i_ under conditions where the membrane is not stretched and the cytoplasmic contents not altered, we used NaN_3_ to lower [ATP]_i_ in intact oocytes. Extracellular application of NaN_3_ (3 mmol/L) was previously reported to lower [ATP]_i_ from 4.6 mmol/L to 1.2 mmol/L and activate heterologously expressed K_ATP_ channels in *Xenopus* oocytes (Gribble et al. 1997). We first confirmed the effect of NaN_3_ on K_ATP_ channels. From a holding potential of 0 mV, voltage ramps of 0.4 s duration were applied from +40 to −80 mV once every 10 s. As reported previously (Gribble et al. 1997), extracellular application of 3 mmol/L NaN_3_ produced a gradual increase in whole cell currents in oocytes co‐injected with *Kir6.2* and *SUR1* cRNAs (Fig. 2A). *I*_KATP_ started to activate within 3–5 min after NaN_3_ application, reached a steady state level after about 10 min and was completely reversible upon washout (Fig. 2A and B). There was an initial transient increase in current level after NaN_3_ removal which could result from relief of direct azide‐induced block of *I*_KATP_ (Gribble et al. 1997). NaN_3_ did not increase currents during the voltage ramps in oocytes not injected with Kir6.2 and SUR1 cRNAs (not shown). Figure 2C compares the average current amplitudes for 10 oocytes recorded at the start (+40 mV) and end (−80 mV) of the voltage ramps before, and after treatment with 3 mmol/L NaN_3_.

**Figure 3.**
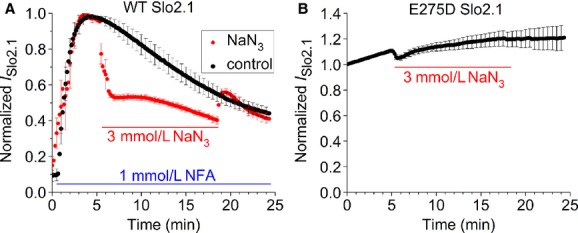
NaN_3_ does not activate Slo2.1 channels heterologously expressed in *Xenopus* oocytes. (A) Plot of time‐dependent effect of 1 mmol/L NFA (●, *n* = 3) or 1 mmol/L NFA co‐applied with 3 mmol/L NaN_3_ (●, *n* = 4) for 13 min (indicated by red bar) on whole‐cell *I*_Slo2.1_ recorded using TEVC from oocytes expressing WT Slo2.1 channels. Currents were recorded during repetitive pulses to 0 mV and normalized relative to the peak current measured in response to NFA (at ~4 min). Oocytes were injected with 1 ng WT *Slo2.1* cRNA and studied 2 days later. (B) NaN_3_ does not enhance currents conducted by constitutively active E275D Slo2.1 channels. Currents recorded during repetitive pulses to 0 mV were normalized relative to the initial current and plotted as a function of time after start of voltage clamp. At 5 min, 3 mmol/L NaN_3_ was added to the bathing solution for a total of 13 min (indicated by red bar), then washed out (*n *= 7). Oocytes were injected with 0.3 ng E275D Slo2.1 cRNA and recorded 2 days later.

Oocytes injected with low amounts of *Slo2.1* cRNA (~1 ng) elicit negligible currents under control conditions. As reported previously (Dai et al. 2010; Garg and Sanguinetti 2012), application of 1 mmol/L NFA to the extracellular solution causes an increase in *I*_Slo2.1_ that reaches a peak in about 4 min, followed by a slow decline over 20 min (Fig. 3A, black circles). The EC_50_ for NFA activation of *I*_Slo2.1_ is 2.1 mmol/L (Dai et al. 2010). We purposely used a non‐saturating concentration of NFA (1 mmol/L) for these experiments to allow for a possible further increase in response to lowering [ATP]_i_. In another group of oocytes, the effect of 3 mmol/L NaN_3_ (in the continued presence of 1 mmol/L NFA) on *I*_Slo2.1_ was determined (Fig. 3A, red circles). After NFA‐mediated activation of *I*_Slo2.1_ reached a peak in 4 min, the control recording solution was switched to a solution that contained 3 mmol/L NaN_3_ plus 1 mmol/L NFA. *I*_Slo2.1_ was rapidly inhibited by NaN_3_ within seconds after its application, and this effect was rapidly reversible upon washout of the NaN_3_ as the magnitude of NFA‐activated *I*_Slo2.1_ was the same at 20–25 min as the control group (Fig. 3A). The rapid inhibition of NFA‐activated Slo2.1 current may result from NaN_3_ competition with NFA binding to the channel, or from direct physical block of the channel by NaN_3_.

E275D Slo2.1 channels are constitutively active in the presence of physiological [Na]_i_ and [Cl]_i_ and absence of NFA (Garg et al. 2013). The magnitude of E275D Slo2.1 channel currents can be increased 2.5‐fold by NFA with an EC_50_ of 192 μM (Garg et al. 2013), indicating that these mutant channels are not fully activated under normal physiological conditions. We examined the effects of NaN_3_ on these channels to address the possibility that activation of WT channels by NFA might somehow prevent inhibition of *I*_Slo2.1_ in response to NaN_3_‐induced reduction in [ATP]_i_. Extracellular application of 3 mmol/L NaN_3_ inhibited these mutant channels to a lesser extent than that observed with WT Slo2.1. E275D *I*_Slo2.1_ slowly increased with time and application of NaN_3_ did not alter this time‐course (Fig. 3B). Thus, unlike K_ATP_ channels, NaN_3_ does not activate *I*_Slo2.1_, indicating a relative insensitivity of Slo2.1 to a decrease in [ATP]_i_.

### Mutation of putative ATP binding site does not alter NFA‐activated *I*_Slo2.1_

**Figure 4.**
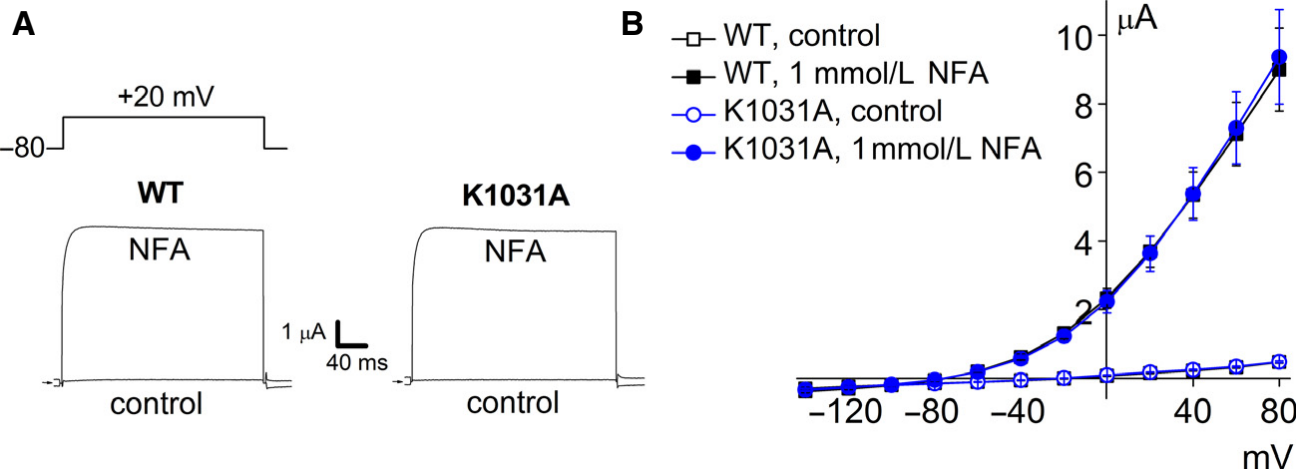
ATP does not inhibit NFA‐activated Slo2.1 channels heterologously expressed in *Xenopus* oocytes. (A) NFA activates WT and K1031A Slo2.1 channel currents measured by TEVC. Under control conditions, only endogenous oocyte currents were measurable. After addition of 1 mmol/L NFA, *I*_Slo2.1_ was activated to a similar extent in oocytes expressing either WT Slo2.1 channel (left panel) or K1031A mutant Slo2.1 channels (right panel). Upper inset depicts voltage‐clamp protocol. (B) Average I–V relationships for WT (□,■) and K1031A (●,○) *I*_Slo2.1_ recorded before and after treatment with 1 mmol/L NFA (*n *= 17 for each channel type). Oocytes were recorded after 2 days of injection with 1 ng WT or K1031A *Slo2.1* cRNA. NFA‐activated currents were larger than control currents (*P *< 0.001; two‐way ANOVA), but not different between WT and K1031A oocytes.

Slo2.1 contains a consensus ATP binding motif (GPKHSGKT) in the C‐terminus of the channel. Mutation of the first Gly to a Ser in this motif was reported to prevent ATP from inhibiting Slo2.1 channel currents (Bhattacharjee et al. 2003). We mutated the highly conserved second Lys of this motif to Ala (K1031A). If as previously proposed, ATP inhibits *I*_Slo2.1_ by binding to this putative binding site, then mutant channels harboring a disrupted motif would be expected to have larger currents than WT channels under similar conditions. We tested this prediction by comparing the magnitude of 1 mmol/L NFA‐activated *I*_Slo2.1_ conducted by WT and K1031A Slo2.1 channels in whole oocytes. Oocytes from a single isolation were injected with 1 ng of WT or K1031A *Slo2.1* cRNA and recorded after 2 days of incubation to achieve comparable levels of expression (Fig. 4A). The I‐V relationships for both currents recorded in the absence and presence of 1 mmol/L NFA are plotted in Fig. 4B. In the absence of NFA, WT and K1031A Slo2.1 channel currents were undetectable and only very small endogenous oocyte currents were measurable (0.47 ±0.03 μA at +80 mV). NFA application provoked a marked and similar increase of current magnitude at all potentials examined between −130 and +80 mV in oocytes expressing WT or K1031 Slo2.1 channels (Fig. 4B). These findings suggest that the putative ATP binding site in the C‐terminus of Slo2.1 is non‐functional.

## Discussion

Due to conflicting reports in the literature, we re‐examined the effects of intracellular ATP on human Slo2.1 K^+^ channels heterologously expressed in *Xenopus* oocytes. Previously, Bhattacharjee et al. (2003) reported that intracellular ATP at a concentration of 5 mmol/L caused >80% inhibition of heterologously expressed human Slo2.1 channels, whereas Berg et al. (2007) reported no inhibition by intracellular ATP of native K_Na_ channels or cloned human Slo2.1 channels. Specifically, neither constitutively expressed Cl^−^‐activated K^+^ currents in striatal interneurons, nor human Slo2.1 heterologously expressed in HEK293T cells measured using the whole cell voltage clamp were inhibited by 3 mmol/L intracellular ATP (Berg et al. 2007). In the present study, we found that application of 5 mmol/L K_2_ATP to the cytosolic side of excised inside‐out macropatches did not inhibit *I*_Slo2.1_, and that mutation of a consensus ATP binding motif did not enhance *I*_Slo2.1_. Unlike the previous studies, we included 10 μM PIP_2_ in the cytosolic solution to prevent run‐down of current and used NFA in most experiments to activate Slo2.1. It is possible that these compounds may have affected the response of Slo2.1 channels to ATP in excised inside‐out patch recordings. To control for this possibility, we showed that lowering [ATP]_i_ with NaN_3_ also did not enhance E275D channel currents. Although these mutant channels are constitutively active, they can be further activated by NFA or elevated [NaCl]_i_ (Garg et al. 2013). Overall, our findings are in agreement with Berg et al. (2007) that intracellular ATP does not inhibit heterologously expressed human Slo2.1 channels.

We performed experiments to determine if lowering [ATP]_i_ by treatment of intact oocytes with NaN_3_ might enhance Slo2.1 channel activity. *I*_Slo2.1_ was recorded from whole oocytes before and after treating oocytes with the metabolic inhibitor, NaN_3_ that inhibits cytochrome C oxidase in the mitochondrial electron transport chain and causes depletion of intracellular ATP (Trapp and Ashcroft 2000). At physiological pH, NaN_3_ is almost completely dissociated in solution and azide ions (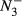) combine with extracellular protons to form HN_3_, a weak acid that can penetrate the plasma membrane (Trapp and Ashcroft 2000). In *Xenopus* oocytes, 10 min exposure to 3 mmol/L NaN_3_ reduced the [ATP]_i_ from 4.6 ± 0.3 mmol/L to 1.2 ± 0.1 mmol/L (Gribble et al. 2000). We confirmed that treatment of oocytes with 3 mmol/L NaN_3_ for 13 min activated heterologously expressed K_ATP_ (Kir6.2/SUR1) channels, but found that the same treatment did not activate either constitutively active E275D Slo2.1 channels or WT Slo2.1 channels partially activated by NFA.

Slo2.1 contains a consensus ATP binding site (1025‐GPKHSGKT‐1032) that is located distal to the second RCK domain in the C‐terminal. The sequence GXXXXGKT, known as Walker motif A (W‐motif) has been shown to bind nucleotides in a wide variety of proteins [e.g. α‐ and β‐subunits of F1‐ATPase, myosin and other ATP‐requiring enzymes (Walker et al. 1982)]. The W‐motif forms a loop around the nucleotide and the highly conserved Lys and Thr residues bind the phosphate group of ATP. An analysis of the crystal structure of 92 proteins that have a W‐motif revealed that in about half of these proteins, the motif did not form a loop or did not bind ATP (Ramakrishnan et al. 2002). Our finding that K1031A Slo2.1 channel currents were of the same magnitude as WT Slo2.1 channel currents indicates that the W‐motif is nonfunctional in Slo2.1.

Together our findings indicate that intracellular ATP does not inhibit human Slo2.1 channels heterologously expressed in *Xenopus* oocytes, consistent with the lack of effect of ATP on these channels when heterologously expressed in HEK293T cells (Berg et al. 2007). This finding implies that during ischemia it is the elevation of [Na^+^]_i_ and [Cl^‐^]_i_, but not the reduction of [ATP]_i_ that causes activation of Slo2.1 K^+^ channels.

## Acknowledgments

The expert technical support of Alison Gardner is gratefully acknowledged.

## Conflict of interest

None declared.
